# Editorial: Plant cell endomembrane dynamics and specialization

**DOI:** 10.3389/fpls.2023.1305963

**Published:** 2023-10-20

**Authors:** Cecilia Rodriguez-Furlan, Lorena Norambuena, Emily R. Larson

**Affiliations:** ^1^ School of Biological Sciences, Washington State University, Pullman, WA, United States; ^2^ Plant Molecular Biology Centre, Department of Biology, Facultad de Ciencias, Universidad de Chile, Santiago, Chile; ^3^ School of Biological Sciences, University of Bristol, Bristol, United Kingdom

**Keywords:** endomembrane, trafficking, autophagy, Rab7, retromer, cell-plate, SNARE, viruses

This special Research Topic highlights current research on the unique characteristics and specialization of the plant endomembrane system. These four reviews and five research articles emphasize the diversity of the molecular and regulatory mechanisms that control endomembrane compartment function, vesicle trafficking, and the dynamics and interactions among the molecules that assist plant cell function, development, and environmental responses.

During plant cell division, the cell plate forms dynamically, involving cytoskeletal reorganization and targeted vesicle trafficking. This process aggregates new cell wall material and membrane components that ultimately fuse with the existing cell walls. In this Research Topic, Shi et al. review the key molecular players involved in this process, like Rab GTPases, tethering complexes, and SNARE proteins that regulate this process. It also explores how these complexes have aided our study of how vesicles and cargo that form cell plates vary from those that traffic to other membranes. Additionally, Allsman et al. introduce a set of maize-specific markers and chemical resources designed for investigating cell plate positioning. Using these tools, the authors show that the delivery of material to the growing cell plate in maize occurs through mechanisms and molecular players divergent from those in Arabidopsis. This discovery is at the forefront of innovative research directions and offers invaluable tools to the scientific community that facilitate further exploration in this field.

While endomembrane protein trafficking pathways have received extensive attention, the specific cargo proteins remain largely uncharted. Li et al. use chemical genomics and proteomics to identify plasma membrane cargoes associated with EXO70A1-mediated secretion in Arabidopsis. EXO70 is one of eight subunits (Sec3, Sec5, Sec6, Sec8, Sec10, Sec15, Exo70, and Exo84) that form the exocyst complex to coordinate the fusion of vesicles with the plasma membrane. Unraveling the molecular components of this process is vital for understanding how plant cells regulate plasma membrane protein delivery, which is crucial for growth, development, and environmental responses.

Another critical aspect of endomembrane traffic involves material delivery to the vacuole. In this Research Topic, Rodriguez-Furlan et al. explore the regulatory function of RAB7 in vacuole traffic during development, biotic interactions, and abiotic stress. The authors provide insight into promising avenues for future research that will elucidate the central role of this endomembrane trafficking regulator. In the path to the vacuole, the retromer complex rescues proteins from degradation. The mini-review by Jha and Larson on the retromer subunit VPS26C, one of three VPS26 proteins in plants but whose function is less defined than other family members. VPS26A and VPS26B participate in canonical retromer pathways that send protein cargo from the trans-Golgi network to late endosomes and the Golgi, while VPS26C may form a complex that sends cargo back toward the plasma membrane, similar to the VPS26C “retriever” complex characterized in animal cells. The authors discuss the potential of a VPS26C-specific retriever complex in plants and highlight unanswered questions like how retromer/retriever complexes are recruited to cell membranes, if these complexes are cell-type dependent, and how VPS26C may function to specialize retromer/retriever pathways in plants.

Within the scope of vacuole-directed trafficking pathways, autophagy is a degradative pathway activated during specific developmental stages and stress responses. Kim et al. report that the FYVE3 proteins interact with the ATG8 isoforms in a phosphatidylinositol-3-phosphate-dependent manner. Although the mode of action of FYVE3 is unknown, the genetic data suggest that it regulates the later stages of autophagosome biogenesis, causing defects in vacuole delivery. This research represents a significant step towards unraveling the intricate processes underlying autophagosome biogenesis and subsequent transport to the vacuole.

The cellular importance of autophagy is largely documented, with its role on plant development and cell differentiation described in Arabidopsis. In this Research Topic, Norizuki et al. demonstrates the role of autophagy during spermatogenesis in the liverwort *Marchantia polimorpha*. The reorganization of plastids is particularly important during spermatogenesis and the authors show that the abolishment of autophagy results in plastid morphology and organization defects. More importantly, Merchantia plastids are degraded in the vacuole via autophagy. These findings demonstrate the importance of autophagy as a key regulator of cell function, with an impact on the development and physiology in liverworts.

The endomembrane system plays a crucial role during stress responses. In their review, Jovanović et al. explore the intricate molecular interactions between virus infection and plant endomembrane-associated pathways. The authors deliver a detailed overview of the numerous viruses that exploit elements of endomembrane pathways for infection, replication, and propagation. The review also explores the multiple endomembrane-related pathways plants use to establish defense pathways in response to infection. This innovative review systematically examines the remarkable progress made in elucidating these molecular interactions.

Endomembrane trafficking is modulated in response to environmental cues, with many SNARE proteins acting as key molecular regulators of vesicle fusion. Salinas-Cornejo et al. describe the molecular function of a SNARE-like protein in *Solanum lycopersicum*, SlSLSP6, whose gene expression is sensitive to salt stress, suggesting a particular function during related environmental stresses in the field. The overexpression of *SlSLSP6* in tomatoes increases salt tolerance, demonstrating endocytosis pathway regulation is related to salt stress responses.

This Research Topic encapsulates the intricacies of the plant endomembrane system and its profound influence on fundamental cellular processes. Together, these contributions enhance our comprehension of plant cell biology, laying the groundwork for future research endeavors and innovations to deepen our understanding of the intricate plant endomembrane trafficking networks.

## Author contributions

CR-F: Writing – original draft, Writing – review & editing. LN: Writing – original draft, Writing – review & editing. ERL: Writing – original draft, Writing – review & editing.

